# Using a Delphi Technique to Seek Consensus Regarding Definitions, Descriptions and Classification of Terms Related to Implicit and Explicit Forms of Motor Learning

**DOI:** 10.1371/journal.pone.0100227

**Published:** 2014-06-26

**Authors:** Melanie Kleynen, Susy M. Braun, Michel H. Bleijlevens, Monique A. Lexis, Sascha M. Rasquin, Jos Halfens, Mark R. Wilson, Anna J. Beurskens, Rich S. W. Masters

**Affiliations:** 1 Research Centre for Autonomy and Participation of people with a chronic illness, Zuyd University of Applied Sciences, Faculty of Health, Heerlen, the Netherlands; 2 Adelante Rehabilitation Centre, Department of Brain Injury, Hoensbroek, the Netherlands; 3 Department of Health Services Research, CAPHRI, School for Public Health and Primary Care, Faculty of Health, Medicine and Life Sciences, Maastricht University, Maastricht, the Netherlands; 4 Centre of Expertise Geriatric Rehabilitation and Chronic Somatic Care, Sevagram Zorgcentra, Heerlen, the Netherlands; 5 Research Centre for Technology in Care, Zuyd University of Applied Sciences, Heerlen, the Netherlands; 6 Adelante Centre of Expertise in Rehabilitation and Audiology, Hoensbroek, the Netherlands; 7 Department of Rehabilitation Medicine, CAPHRI, School for Public Health and Primary Care, Faculty of Health, Medicine and Life Sciences, Maastricht University, Maastricht, the Netherlands; 8 Department of Sport and Health Sciences, University of Exeter, Exeter, United Kingdom; 9 Department of Family Practice, CAPHRI, School for Public Health and Primary Care, Faculty of Health, Medicine and Life Sciences, Maastricht University, Maastricht, the Netherlands; 10 Institute of Human Performance, University of Hong Kong, Hong Kong, China; 11 Department of Sport and Leisure Studies, University of Waikato, New Zealand; University of Münster, Germany

## Abstract

**Background:**

Motor learning is central to domains such as sports and rehabilitation; however, often terminologies are insufficiently uniform to allow effective sharing of experience or translation of knowledge. A study using a Delphi technique was conducted to ascertain level of agreement between experts from different motor learning domains (i.e., therapists, coaches, researchers) with respect to definitions and descriptions of a fundamental conceptual distinction within motor learning, namely implicit and explicit motor learning.

**Methods:**

A Delphi technique was embedded in multiple rounds of a survey designed to collect and aggregate informed opinions of 49 international respondents with expertise related to motor learning. The survey was administered via an online survey program and accompanied by feedback after each round. Consensus was considered to be reached if ≥70% of the experts agreed on a topic.

**Results:**

Consensus was reached with respect to definitions of implicit and explicit motor learning, and seven common primary intervention strategies were identified in the context of implicit and explicit motor learning. Consensus was not reached with respect to whether the strategies promote implicit or explicit forms of learning.

**Discussion:**

The definitions and descriptions agreed upon may aid translation and transfer of knowledge between domains in the field of motor learning. Empirical and clinical research is required to confirm the accuracy of the definitions and to explore the feasibility of the strategies that were identified in research, everyday practice and education.

## Background

Motor learning is a central issue in sports, but has recently received increased attention in the context of rehabilitation [1]–[6]. A departure point for explanatory models of motor learning, in a variety of healthy and rehabilitation target groups, is the broad distinction between conscious and non-conscious attributes of the motor learning process. The distinction is often delimited by an implicit-explicit conceptualisation first made popular in cognitive psychology [7], which proposes that implicit motor learning targets more non-conscious attributes of the motor learning process, whereas explicit motor learning targets more conscious attributes of the motor learning process [8], [9].

Although investigation of implicit and explicit forms of motor learning has become common-place in recent years, particularly in the sport-related motor literature [9], [10], for the most part, terminology has been insufficiently uniform. This has raised a barrier that hampers exchange of knowledge within and between fundamental domains of research and practical target groups.

For example, in describing or defining implicit and explicit (motor) learning, independent research groups have focused on the type of knowledge accrued during the learning process, e.g. [8], the amount of attention or awareness needed to learn, e.g. [11]–[13], or the way the results of the learning process are measured, e.g. [11], [14]. The terms implicit and explicit are sometimes also used to refer to the underlying memory systems that are involved, e.g. [15], as synonyms associated with declarative and procedural knowledge, e.g. [12] or to describe the actual application of learning in practice, e.g. [16]. However, there is some overlap in underlying conceptualisations of implicit and explicit motor learning. For example, in many definitions and descriptions explicit motor learning is associated with conscious cognitive processes, whereas implicit learning is associated with non-conscious cognitive processes, e.g. [8], [17]. The lack of agreement sometimes results in different, or even conflicting, applications of implicit and explicit learning in study paradigms, clinical practice and education.

Intervention strategies, such as discovery learning, analogy learning and errorless learning, have been used to shape the motor learning process in clinical or non-clinical studies within different target groups [18]–[20]. In general, intervention strategies that lead to high conscious awareness of how the motor behavior is accomplished are applied to promote explicit motor learning, whereas intervention strategies resulting in low conscious awareness of how the motor behavior is accomplished are applied to promote implicit motor learning [8], [9].The theoretical underpinning for this implicit/explicit distinction proposes that motor learning is a process in which solutions to the motor problem are discovered either explicitly through a process of hypothesis testing made possible by the human ability to temporarily manipulate and store information consciously in working memory, or implicitly through a process of discovery that does not rely on conscious manipulation and storage of information by working memory [8], [9], [21]. For example, reducing the amount of errors made during the motor learning process (errorless or error-reduced learning) is thought to moderate the need for hypothesis testing about possible motor solutions, which minimizes working memory involvement in movement and promotes implicit motor learning [19], [22]. Further, it has been argued that learning a motor task while performing a concurrent cognitive task (dual task learning) prevents working memory from temporarily storing conscious information related to motor solutions because working memory must engage in completing the cognitive task. Thus, the motor behaviour is learned more implicitly than if a cognitive secondary task was not performed concurrently [8].

However, not all intervention strategies are used unambiguously with respect to the implicit/explicit distinction. For many motor learning strategies, it seems unclear whether they promote implicit or explicit motor learning or whether their ability to promote either form of learning is a function of the target population or the specific learning context in which they are applied. For example, discovery learning is regarded by some researchers as likely to result in predominantly implicit learning outcomes, whereas, other researchers argue that predominantly explicit outcomes result [23]. Yet, trial and error learning, which in practice seem little different from discovery learning, has been described to promote explicit motor learning [19], [24].

For therapists, coaches, researchers and teachers, uniform terminology is particularly important. Effective transfer of research results to clinical practice and education is promoted by clear terminology, and allows therapists and coaches to speak a common language among themselves (e.g., to set up treatment plans) or when instructing students [25]. The aim of this study was therefore to seek consensus regarding the definitions, descriptions and classification of terms related to the general distinction between implicit and explicit forms of motor learning.

## Method

A survey consisting of a series of sequential rounds interspersed by controlled feedback [26] was performed to collect and aggregate informed judgments about motor learning from a group of experts. The survey consisted of three rounds, which were designed and distributed using an online survey programme (SurveyMonkey Inc, SurveyMonkey.com, California, USA). More detailed information about the method and rationale for the entire survey is presented elsewhere [27].

A Delphi technique was embedded into the first two rounds of the survey to seek consensus regarding definitions, descriptions and classifications related to the explicit/implicit distinction in motor learning. Although there is minimal scientific evidence available to inform decisions about the number of survey rounds appropriate for a Delphi technique, two or three rounds have typically been employed [28]. Information regarding the content and the results of the third round is not presented here as this round was not used as part of the Delphi technique.

The Central Ethics Committee Atrium-Orbis-Zuyd (Institutional Review Board) was contacted and formal written permission to perform the study described in the protocol [26] was obtained (13-N-144). The study was excluded from IRB review, because under the law, Medically Scientific Research with people (WMO), it does not submit people to actions or impose specific behaviors on them.

### Procedure

A referee group consisting of seven researchers with backgrounds in epidemiology, physiotherapy, occupational therapy, movement sciences and psychology supervised and monitored the process. The group conducted the literature search, identified experts to be approached to complete the surveys, and prepared the questions for each survey round. Between each survey round, the group performed a preliminary analysis of data blinded to the identity of the experts. In addition, two members of the referee group (MK, SB) were responsible for distributing and monitoring the survey (e.g., sending reminders and feedback reports).

A panel of international experts was invited to contribute to the study. Panel members were initially selected on the basis of literature search or the networks of the referee group. Criteria for selection of an expert were based on either scientific publication(s) in the field of motor learning (researcher) or at least three years of working experience applying motor learning in practice plus involvement in education or research (therapist, coach, lecturer).

In a preliminary recruitment round, eligible experts were invited by mail to participate in the study. They were given a comprehensive introduction to the aims and content of the survey rounds, informed of the expected amount of time necessary to complete each survey and provided with a personal link to the online survey program. After informed consent was obtained from the experts, they were asked to provide personal information (e.g., background, years of experience, special interests). Those experts who were invited to participate were asked to recommend other experts in the field (so-called snowball sampling method), who were subsequently contacted in the same manner.

No clear guidelines regarding the optimal panel size for a Delphi study exist [29]. Consistent with another study using a Delphi technique [30], a minimum panel size of 30 experts was targeted, comprising approximately ten motor learning researchers in rehabilitation, ten in healthy individuals and sports and ten with experience applying motor learning in daily practice.

#### Content of the survey rounds and analysis

Within the survey rounds, we distinguished between definitions and descriptions. The term “definition” was used when referring to forms of learning (e.g., implicit, explicit), whereas the term “description” was used when referring to motor learning strategies (e.g., errorless learning, trial and error learning). We made this distinction because the term ‘definition’ implies theoretical attributes/features of learning, while the term ‘description’ implies elements of how a strategy is applied.

Additionally, implicit and explicit motor learning have been described as representing a dichotomy in learning and also as representing tail ends of a learning continuum, so for the categorisation of intervention strategies we used answering categories that left room for both perspectives.

Table 1 presents the content of the survey rounds. Each survey round was divided into two parts. The first part of Round 1 focussed on creating a basic definition of implicit and explicit motor learning. The second part focussed on identifying, describing and classifying learning strategies. Questions in part one of Round 2 and 3 were used to verify responses in the first round and second round respectively and to elaborate issues identified by the expert panel. Questions in part two of Round 2 and 3 addressed other predefined topics (results are not presented in this article).

**Table 1:** Content and structure of the survey rounds.

Round	Content	Questions	Answering options
**1**	**Definition of implicit and explicit learning (part 1)**: Experts were provided with attributes used in the literature to define or describe implicit and explicit motor learning. The following questions were asked:	*The definition of implicit motor learning should in your opinion definitely contain the following attributes:*	multiple choice, more answers possible, see Results section for an overview of attributes provided
			
			
		*The definition of explicit motor learning should in your opinion definitely contain the following attributes:*	multiple choice, more answers possible, see Results section for an overview of attributes provided
		*If you are aware of a definition of explicit/implicit motor learning from the literature, with which you can agree (best option), please give a citation of this definition in the box below. Please include the reference.*	open comment box
	**Identification and description of strategies promoting motor learning and their classification (part 2)**: A list of motor learning strategies was provided together with a description based on the literature. For each strategy the following questions were asked (see Results section for an overview of strategies provided):	*Do you know the strategy?*	dichotomous choice: yes/no, experts who agreed were referred to the next question, expert who did not agree were referred to the next strategy in the list
		*Do you agree with the provided description? If not, please indicate what is missing or incorrect and/or provide your ideal description.*	dichotomous choice: yes/no and open box, see results section for an overview the provided descriptions
		*How would you classify the strategy?*	multiple choice, only one answer possible, see results section for the answering categories provided
		*Have you used the strategy before (research or practice)?*	dichotomous choice: yes/no
		*Can you give an example of how you would apply this strategy in practice or research?*	open comment box
***2***	**Confirmation of results of Part 1 from Round 1**: Based on the results of part 1, the definitions of implicit and explicit motor learning were provided. Separately for the definitions of implicit and explicit motor learning the experts were asked:	*Do you, in general, agree with the definition?*	dichotomous choice: yes/no
		*Please state any comments or additional information in the box.*	open comment box
	**Confirmation of results Part 2 from Round 1**: Only the best known-strategies were taken into account in this round. For each strategy the experts were asked:	*Do you, in general, agree with the modified description?*	dichotomous choice: yes/no

In preparation of the Delphi, the referee group performed a literature search in different fields of motor learning (sports, rehabilitation, fundamental research) using both scientific research articles and grey literature. The group identified several search terms, implicit, explicit, motor learning, skill acquisition, and used MeSH terms when possible. From these resources, e.g. [1], [8], [11], [17], [31]–[35], statements which were related to implicit and explicit motor learning were extracted and compared. The referee group tried to improve readability of the statements by using comparable formulation.

Analysis of the data was conducted blind to the names and characteristics of the expert respondents. Open comments and additions made by the experts were clustered in themes and carefully considered by the referee group. Consistent with other studies, consensus was considered to have been reached when ≥70% of the experts agreed on a certain topic [30], [36], [37]. Differences in values and beliefs within the different professions represented by the experts might have influenced the results, so in cases where ≥70% of the experts agreed, the referee group checked for a profession-based imbalance in the responses, which was not the case.

If consensus was achieved, final definitions and descriptions were formulated. If no consensus was achieved, the topic and answers were presented to the expert panel in the following round.

After each round, panel members received a feedback report that summarised the response percentages for each question, as well as responses to open questions and additional comments. In these feedback reports, the results were clustered but not analysed or interpreted.

## Results

**Figure 1:**
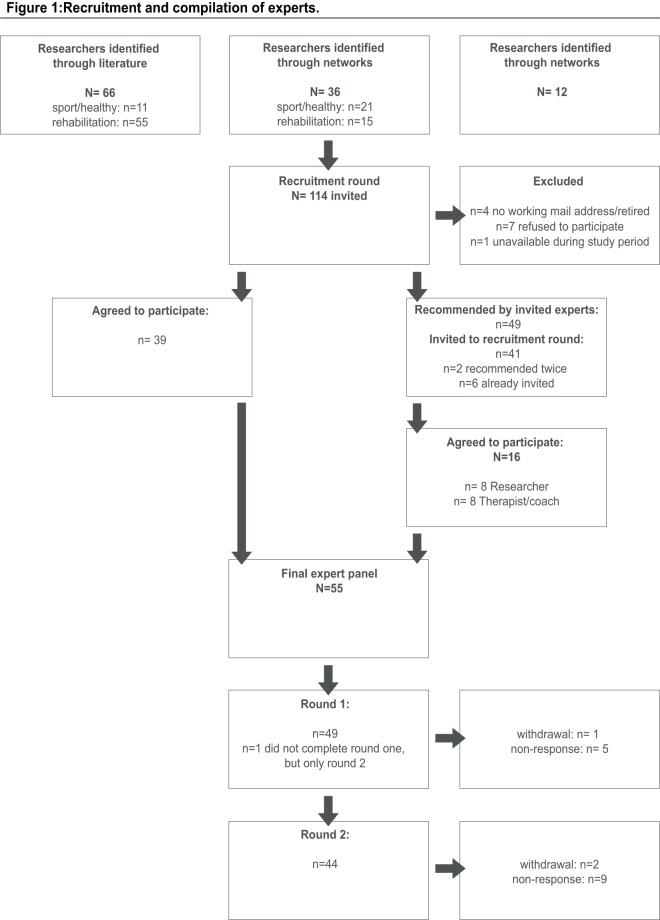
Recruitment and compilation of experts.

The recruitment process is shown in Figure 1. In total, 155 experts were invited to participate. Thirty-nine experts agreed to participate initially and recommended a further 49 experts. Forty-nine experts completed the Round 1 survey and 44 completed the Round 2 survey. Characteristics of these experts are shown in Table 2. Experts were heterogeneous with regard to age, background and current working situation. Although the expert panel was internationally diverse, most were based in Europe. Of the 11 experts who did not respond to invitations or reminders, only two reported lack of time as the reason for non-response.

**Table 2:** Characteristics of the expert panel.

Category	Subcategory	Results (absolute numbers)
**Gender**	Male	23
	Female	27
**Age category**	20–30:	3
	30–40:	12
	40–50:	19
	50–60:	14
	60–70:	2
	>70:	2
	Not wanted to state/missing:	3
**Working country**	England/UK:	16
	The Netherlands:	8
	USA:	6
	Australia:	4
	Canada:	4
	France:	2
	Belgium:	2
	Germany:	2
	China/Hong Kong:	1/2
	New Zealand:	1
	Switzerland:	1
	Missing:	1
**In last 5 years mainly worked as**	Researcher:	22
	Lecturer/Educator:	8
	Therapist:	11
	Both researcher and lecturer equally:	2
	Other (e.g., consultant, psychologist):	6
	Missing:	1
**Background** *	Rehabilitation Practitioner (PT, OT, ST^#^):	25
	Movement Scientist:	18
	Psychologist:	11
	Coach:	8
	Other (e.g., biomechanist, sport scientist):	5
**Expert in which motor learning area** *	Rehabilitation:	35
	Sports:	18
	Fundamental research (neuroscience):	13
	Elderly:	9
	Children:	4
	Education:	2
	Other (e.g., cognitive psychology, mental health):	4
**Target population working with** *	Neurological patients (adults):	23
	Elderly:	14
	Healthy population in general:	12
	Athletes:	11
	Neurological patients (children):	8
	Orthopaedic patients (adults):	1
	Healthy children:	1
	Other (e.g., therapists, patients with mental health problems):	5
**Years of experiences**	Research	Mean: 14.1 (SD: 11.8)
	Not applicable	7
	Practice	Mean: 11.8 (SD: 10.0)
	Not applicable	10

*:more answer options were possible;^#^PT: Physiotherapist, OT: Occupational Therapist, ST: Speech and Language Therapist; Table is based on data of n = 50 experts (n = 49 experts completed Round 1 and 2; n = 1 expert completed Round 2 only);

### Definitions of implicit and explicit motor learning

The results with regard to the definitions of explicit and implicit motor learning are shown in Tables 3 and 4. Of the experts, 95.5% agreed in general with the following definition of explicit motor learning: *learning which generates verbal knowledge of movement performance (e.g., facts and rules), involves cognitive stages within the learning process and is dependent on working memory involvement*.

**Table 3:** Definition of explicit motor learning.

Explicit Motor Learning	
**Round 1 (n = 49)**	
**Attributes provided in**	**•Involves cognitive stages:** * **85.7%**
**first round and**	**•Generates verbal knowledge of movement performance (e.g., facts and rules): 79.6%**
**percentages chosen**	**•Dependent on working memory involvement: 73.5%**
	**•**Facilitated by instructions about how to perform the movement: 67.3%
	**•**With intention to learn: 63.3%
	**•**With purposeful hypothesis testing: 42.9%
	**•**Learning processes are faster (compared to implicit): 22.4%
	**•**Other: 12.2%
**Round 2 (n = 44)**	
**Definition provided in second round**	**Explicit motor learning can be defined as learning which generates verbal knowledge of movement performance (e.g., facts and rules), involves cognitive stages within the learning process and is dependent on working memory involvement**
**% agreement**	**95.5%**
**Comments after second**	•Aspect of an ‘internal focus’ should be involved (n = 0/1)
**round**	•Three key attributes in definition are related and therefore redundant (n = 1/0)
**(n = agreed/disagreed)^#^**	•Disagreement about the involvement of cognitive stages (n = 2/0)/working memory (n = 2/0)
	•Disagreement about the distinction between implicit and explicit learning in general (n = 1/0)
	•Disagreement about the verbal/explicit instructions (n = 2/0)

*Attributes in **bold** were taken into account for the definition in Round 2; #:Comments of experts who did not agree are underlined. Numbers in brackets signify amount of times that this comment was provided by experts who agreed/disagreed with definition.

**Table 4:** Definition of implicit motor learning.

Implicit Motor Learning	
**Round 1 (n = 49)**	
**Attributes provided in**	**•No or minimal increase in verbal knowledge** * **: 81.6%**
**first round and**	**•Skills are (unconsciously) retrieved from implicit memory: 81.6%**
**percentages chosen**	**•Skills are learned without awareness: 69. 4%**
	**•**Without exposure to verbal instructions about how to perform: 53.1%
	**•**Robust to disruption: 42.9%
	**•**Learning process takes longer (compared to explicit): 42.9%
	**•**Without an initial cognitive stage: 36.7%
	**•**No purposeful hypothesis testing: 30.6%
	**•**Other: 16.3%
**Round 2 (n = 44)**	
**Definition provided in second round**	**Implicit motor learning can be defined as learning which progresses with no or minimal increase in verbal knowledge of movement performance (e.g., facts and rules) and without awareness. Implicitly learned skills are (unconsciously) retrieved from implicit memory.**
**% agreement**	**88.6%**
**Comments after second**	•Disagreement about the fact that skills are learned without awareness (n = 7/2)
**round**	•Disagreement about use of the term “implicit memory” (n = 5/1)
**(n = agreed/disagreed)^#^**	•Same attributes should be used in definition of implicit and explicit (n = 1/0)
	•Definition contains assumptions that should be tested first (n = 0/1)
	•Definitions should take the complexity of cognitive involvement more into account (n = 1/0)
	•External focus should be involved (n = 1/1)

*Attributes in **bold** were taken into account for the definition in Round 2. #: Comments of experts who did not agree are underlined. Numbers in brackets signify amount of times that this comment was provided by experts who agreed/disagreed with definition.

Six experts (12.2%) proposed additional attributes of the definition of explicit learning that were not provided initially (category “other”). The additions included practical attributes (e.g., “*using an internal focus of attention*”, “*using feedback*”) and attributes related to the result of explicit learning (e.g., “*responsible for rapid early improvement*”), but they were not incorporated into the definition as each was only suggested by one or two experts.

Of the experts, 88.6% agreed in general with the following definition of implicit motor learning: learning which progresses with no or minimal increase in verbal knowledge of movement performance (e.g., facts and rules) and without awareness. Implicitly learned skills are (unconsciously) retrieved from implicit memory.

Eight experts (16.3%) proposed additional attributes of the definition of implicit motor learning, which were eventually not incorporated into the definition, despite their importance for discussion (e.g., an “*external focus of attention*” is used; the learning is “*goal orientated*”; relies on “*functional practice in a meaningful environment*”). One expert pointed out that implicit learning is “*not non-cognitive*” and “*not unconscious*” but rather “*non-verbal*”. This expert further pointed out that implicit learning “*often does involve awareness of trying to accomplish something*”.

Thirteen existing definitions or extractions from literature were preferred or deemed to be as good as the new definitions, but none were mentioned more than once.

### Descriptions of strategies

Table 5 provides an overview of 10 motor learning intervention strategies that were identified from the literature and gives the percentage of experts who indicated that they were aware of the strategies and/or used the strategies. Seven strategies were known by more than 70% of the experts and were therefore included in Round 2. After modification of the strategy descriptions, based on the comments in Round 1, consensus was achieved for six of the seven descriptions (see Table 6). Only for *observational learning* did percentage agreement decrease slightly after the description was reformulated (from 69.4% to 68.2%).

**Table 5:** Percentage of experts who knew/used the provided strategies.

Strategy*	Percentage of experts who knew the strategy	Percentages of experts who have used the strategy before in research or practice
**Trial and error**	**91.8%**	73.5%
**Observational**	**89.8%**	67.3%
**Errorless**	**89.8%**	63.3%
**Movement imagery**	**85.7%**	40.8%
**Discovery**	**77.6%**	36.7%
**Dual task**	**77.6%**	57.1%
**Analogy**	**73.9%**	55.1%
Incidental	65.3%	
Self-regulatory	49.0%	
Constraints-led approach	46.7%	

*Strategies **in bold** were taken into account in Round 2.

**Table 6:** Description of the best known strategies.

	Round 1 (n = 49)			Round 2 (n = 43/44)^#^	
	Description provided in first round	% agreement	Comments*	Adapted description in second round	% agreement
**Trial and error learning**	*Learning by repeatedly attempting to perform a task during which errors are detected and corrected.*	71.4%	**The learner must (be able) to detect the error (n = 6)**; Learning is an iterative process (n = 1); Correction of errors should not be emphasised (n = 1)	*Learning by repeatedly attempting to perform a task during which the learner detects errors and corrects them.*	84.1% agreed; 9.1% preferred description from Round 1
**Observational learning**	*Learning by observing a movement. The observer determines the key spatial and/or temporal features of the task through observation, thereby creating a cognitive representation of the action pattern.*	69.4%	**Unsure about/delete “cognitive representation”(n = 7); The demonstrator/therapist can also direct the learner to the key features (n = 1)**	*Learning by observing a movement. The observer determines the key spatial and/or temporal features of the task through observation, and/or is directed to these features by the demonstrator/therapist.*	68.2% agreed; 20.5% preferred description from Round 1
**Errorless learning**	*Learning facilitated by constraining the learning environment so that very few errors occur.*	67.3%	**Learning environment and the instructions and skill difficulty can be constrained as part of the learning environment (n = 3)**; Should be applied particularly in early phase of learning (n = 1); Replace “very few errors” with “no errors” (n = 1)	*Learning facilitated by constraining the learning environment (e.g., instructions, skill difficulty) so that very few errors occur.*	77.3% agreed;13.6% preferred description from Round 1
**Movement Imagery**	*Learning by imagining oneself undertaking the skilled movement without actually doing the movement.*	71.4%	**Imagery should be from the first person perspective (n = 2); Exchange “undertaking” with “performing” (n = 1)**; Suggestions for terming the strategy (mental rehearsal, motor imagery) (n = 2)	*Learning by imagining oneself performing the skilled movement (in the first or third person perspective) without actually physically performing the movement.*	81.8% agreed; 13.6% preferred description from Round 1
**Discovery learning**	*Learning without guidance, instructions or feedback from another person.*	57.1%	**Without information from other sources (book, website) (n = 2)**; It is necessary to give instructions or feedback (n = 2); Learning is facilitated by (constrained) context (n = 3); Use (pure) discovery learning as a synonym for Trial and error (use this description) (n = 1)	*Learning without guidance or feedback from another person or information source.*	75.0% agreed; 18.2% preferred description from Round 1
**Dual-task learning**	*Learning of a skill during simultaneous performance of another skill. The secondary task can be a motor or cognitive task.*	61.2%	**The (second) task must be of equal importance/difficulty and attention demanding (n = 5)**; Doubts about whether dual task is a form of learning (n = 3)^+^	*Learning of a skill while simultaneously performing another task. The second task can be a motor or cognitive task but must be attention demanding*	81.8% agreed; 9.1% preferred description from round one
**Analogy learning**	*Learning facilitated by metaphors. The complex structure of the to-be-learned skill is integrated in a simple biomechanical metaphor that the learner is provided with*	51.0%	Did not agree with term ‘biomechanical’ (n = 6)	*Learning facilitated by metaphors. The complex structure of the to-be-learned skill is integrated into a simple metaphor that the learner is provided with.*	95.5% agreed; 2.3% preferred description from round one

*Comments in **bold** were taken into account for the adapted description; ^#^One expert did not complete all questions; ^+^This remark was taken into account in separate questions in Round 2 (results not presented).

### Classification of strategies

Responses regarding classification of whether the strategies are likely to result in (more) implicit or (more) explicit forms of motor learning were diverse (see Table 7).

**Table 7:** Classification of the learning strategies.

Classification	Trial and error learning (n = 44)	Observational learning (n = 44)	Errorless learning (n = 44)	Movement imagery (n = 42)	Discovery learning (n = 38)	Dual task learning (n = 38)	Analogy learning (n = 36)
Implicit	2.3%	6.8%	18.2%	7.1%	18.4%	26.3%	11.1%
More implicit than explicit	9.1%	20.5%	45.5%	19%	26.3%	47.4%	58.3%
Both implicit and explicit	25%	34.1%	15.9%	21.4%	23.7%	7.9%	5.6%
More explicit than implicit	25%	25%	4.5%	23.8%	18.4%	10.5%	5.6%
Explicit	29.5%	9.1%	4.5%	11.9%	5.2%	0%	11.1%
Other	9.1%	4.5%	11.4%	16.7%	7.9%	7.9%	8.3%

Round 1 suggested that none of the strategies can be categorized as promoting just one form of motor learning. For the errorless, dual-task and analogy learning strategies, there was however a slight trend for the experts to consider these strategies as likely to result in a more implicit form of learning. Depending on the strategy, between 1 and 5 experts did not classify the separate strategies into one of the provided categories, but chose the option ‘other’ (between 4.5–16.7% of the sample).

A common argument in the open comment box was that the strategy could promote both implicit and explicit motor learning. Factors, such as, instructions, constraints in the environment, type of task/skill and the abilities of the learner were all deemed to have an influence on the outcomes of the learning strategy. According to the experts, manipulation of these factors has a profound influence over the degree to which a strategy results in implicit or explicit motor learning.

### Additional strategies

Twenty-two alternative motor learning strategies were suggested, which were not included in the initial list presented to the experts (e.g., win shift lose stay, verbal overshadowing, blocked practice, applied behaviour analysis). None of these strategies were mentioned by more than one expert and were therefore not incorporated in the following surveys rounds. Other suggestions were related more generally to the focus of attention during learning, the provision of feedback, the repetition and variability of practice and manual facilitation.

As a result of the diversity in answers and additional statements made by the experts, the referee group decided not to strive to seek consensus with regard to the classification but rather to explore the reasons for diversity. This was done in the third survey round and resulted in an overview of practical experiences, opinions and verifications of statements (results not presented).

### Examples of the application of the strategies in clinical practice

The number of examples of the application of different strategies in clinical practice ranged from 30 (discovery learning) to 41 (errorless learning). For each strategy, the referee group chose two examples to present in this article (Table 8).

**Table 8:** Examples of the best known strategies provided by the experts.

Strategy	Two random selected examples provided by experts
Trial and error learning	*“Structure the learning environment so that errors will be made, but a positive outcome is achievable. Inform the learner that following the practice session they will be asked to describe the different techniques they tried and list what worked and what didn't.”*
	*Putting on a jumper: “Prompt when needed to avoid frustration but encourage patient to do without help. Positive reinforcement. Requires good attention levels.”*
Observational learning	*“Demonstration is probably used quite frequently by therapists who wish to demonstrate what they want a patient to do, or how they want them to do it. In my experience, this is generally accompanied by verbal instructions, making it more explicit. Patients may observe each other in a group setting, which could be formally set up (working in pairs) to create an observational learning environment –for example, for performing balance tasks.”“*
	*“This technique is frequently used in dance classes where one dancer acts as a model and the other observe and then imitate.”*
Errorless learning	*“In aikido, novices may learn new techniques with a more experienced partner that would help novices to succeed every time they perform it.”*
	*“Learning to walk after a stroke with body weight support and a treadmill, and gradually increasing the body weight the person is taking as well as the treadmill speed.”*
Movement Imagery	*“With patients who are physically unable to perform such a movement at the beginning of rehabilitation, or if they fatigue quickly during physical practice.”*
	*“Imaging oneself climbing a wall and then climbing it.”*
Discovery learning	*“Children in a playful setting discover biomechanics of building with blocks.”*
	*“For teaching previously unknown skill – e.g., making piece of toast one handed. Explain what is needed and leave patient to work out how. Would need high level problem solving including attention and memory. Avoid distraction. Would require positive reinforcement.”*
Dual task learning	*“Having a child count backwards by 2's (depending on age and cognitive level) while walking on the balance beam.”*
	*“Clinicians working on more complex or real-world environments where motor tasks are combined with other motor tasks or cognitive tasks (such as talking). Instructions can be used to prioritize a task or it can be left to the discretion of the performer. Feedback and measures of performance should be provided on both tasks.”*
Analogy learning	*“Jumping pattern: "reach for an apple up in the tree” “Basketball shot: "putting your hand into the cookie jar."*
	*“Dance tango (in particular how to provide a good abrazo): like maintaining a newspaper always opened.”*

## Discussion

The aim of this study was to seek consensus on the definitions and descriptions of terms related to the conceptual distinction between implicit and explicit motor learning. Within a heterogeneous international group of experts, consensus regarding definitions of implicit and explicit motor learning, and descriptions of the best known strategies used in the context of implicit and explicit motor learning, was reached. Both definitions incorporate central aspects of motor learning (e.g., form of memory and type of knowledge). Incorporation of more than one central aspect of motor learning is preferable to outlining a single aspect, as sometimes occurs in definitions, and suggests that there was at least some degree of consensus by the experts.

Consensus suggests that experts from the different fields represented within the study may think about and describe motor learning and the underlying processes in a comparable way, at least at a more theoretical level.

According to the responses of the experts in this study analogy learning, errorless learning and dual task learning seem to promote more implicit learning in general. However, no consensus was reached within the expert panel on the classification of motor learning strategies for promoting a (more) implicit or (more) explicit form of motor learning. Based on the results of this study, it seems that most intervention strategies do not naturally promote implicit or explicit motor learning. They can promote either form of learning depending on their use in a specific learning situation and/or target population. It is probably impossible, and perhaps not even desirable, to achieve consensus. This result might be a consequence of the complexity of applying motor learning strategies in everyday practice. For example, athletes or patients usually need a tailored approach and the application of learning strategies is determined by multiple factors. Consequently, for research and education it is even more important that the application of an intervention strategy in a specific context is always described in detail.

### Critical reflection on the study and the study results

To our knowledge, this is the first study that uses a Delphi technique in the field of motor learning. The results generated and summarized within the study are based on knowledge, opinions and practical experiences of an international expert panel. Consequently, the results should be interpreted tentatively; an expert group's opinion rather than empirical evidence.

Importantly, although consensus was obtained regarding the definitions, this does not mean that all of the experts, or indeed the authors (referee group), agree with the final definitions and descriptions. For instance, the final definition of implicit motor learning suggests that learning progresses “without awareness”, but there are clearly occasions (e.g., sport, rehabilitation) when a person has intention to learn and is aware of learning, especially when outcome feedback is readily available [21], [38]. It also seems unlikely that learning ever progresses with ‘no’ increase in verbal knowledge.

Although the Delphi technique is a well-accepted method for investigating opinions, there is currently no agreement on the meaning of *consensus* [28]. In our study, consensus was regarded as agreement within a selected group of leading experts on a certain topic, based on a criterion of 70% agreement or greater. However, lack of consensus (i.e., less than 70% agreement) does not directly imply that a statement was invalid, but may suggest that more plausible possibilities exist or that no alternatives exist yet. Numerous comments and statements were made by individual experts in response to the open questions. Although all were distributed in the feedback report, most were not carried back into the survey. As the aim of the study was to achieve consensus (a quantitative approach), we unfortunately were not able to take all single statements into account (a more qualitative approach). Consequently, comments/statements which other experts may have agreed upon might have been overlooked.

The quality of the findings from a Delphi study is strongly related to the heterogeneity and representativeness of the expert panel. Although the response rate in the current study was low, the experts who participated can be described as heterogeneous with regard to their backgrounds, special interest and working experience. Further, the different practical areas of motor learning are represented by the expert panel. We tried to overcome selection bias within the sample by using snow-ball sampling; nevertheless, some selection bias may have occurred, as most of the experts who participated were based in Europe. This might be explained by the fact that six of the seven referee group members, whose networks were used to identify experts, were also based in Europe. We do not know to what extent the origin and background of the experts influenced the results, so we acknowledge that cultural values may account for some of our findings (especially, lack of consensus).

### Contribution to scientific literature and implications for research

Despite the limitations already discussed, we believe that it is important to use uniform terminology when describing the content of motor learning studies and practical sessions, and within education. The study is a first important step towards helping therapists, researchers and other professionals to communicate about motor learning in general and to distinguish fundamentally between implicit and explicit motor learning more specifically. The added value of the study is that the definitions and descriptions that emerged are based on the opinions of an expert panel from different fields of motor learning, which might help to promote a common language across different fields.

Future applied research is needed to confirm the findings. Underlying neurophysiological and behavioural aspects of the definitions should be investigated by fundamental research. Clinical research investigating clearly defined and described techniques is needed to investigate whether the definitions of implicit and explicit motor learning, as well as the descriptions of the strategies, are feasible and applicable within clinical practice.

## References

[1] van Halteren-van Tilborg IA, Scherder EJ, Hulstijn W (2007) Motor-skill learning in Alzheimer's disease: a review with an eye to the clinical practice. Neuropsychol 17: 203–12. 10.1007/s11065-007-9030-1 PMC2039835 17680369

[2] Abbruzzese G, Trompetto C, Marinelli L (2009) The rationale for motor learning in Parkinson's disease. Eur J Phys Rehabil Med 45: 209–14. 19377414

[3] van Tilborg I, Hulstijn W (2010) Implicit motor learning in patients with Parkinson's and Alzheimer's disease: differences in learning abilities? Motor Control 14: 344–61. 20702895 10.1123/mcj.14.3.344

[4] Dechamps A, Fasotti L, Jungheim J, Leone E, Dood E, et al (2011) Effects of different learning methods for instrumental activities of daily living in patients with Alzheimer's dementia: a pilot study. Am J Alzheimers Dis Other Demen 26: 273–81. 21502092 10.1177/1533317511404394 PMC10845318

[5] Kwakkel G, Kollen B, Lindeman E (2004) Understanding the pattern of functional recovery after stroke: facts and theories. Restor Neurol Neurosci 22: 281–99. 15502272

[6] Kitago T, Krakauer JW (2013) Motor learning principles for neurorehabilitation. Handb Clin Neurol 110: 93–103. 23312633 10.1016/B978-0-444-52901-5.00008-3

[7] Reber AS (1967) Implicit learning of artificial grammars. J Verbal Learning Verbal Behav 6: 855–63.

[8] Masters RSW (1992) Knowledge, knerves and know-how: the role of implicit versus explicit knowlegde in the breakdown of a complex motor skill under pressure. Br J Psychol 83: 343–56.

[9] Masters RSW, Poolton J (2012) Advances in implicit motor learning. In: Hodges NJ, Williams AM, editors. Skill Acquisition in Sport: Research, Theory and Practice. 2nd ed. London: Routledge. pp. 59–75.

[10] Williams AM, Hardy L, Mutrie N (2008) Twenty-five years of psychology in the Journal of Sports Sciences: A historical overview. J Sports Sci 26: 401–412. 18228168 10.1080/02640410701765631

[11] Boyd LA, Winstein CJ (2003) Impact of explicit information on implicit motor-sequence learning following middle cerebral artery stroke. Phys Ther 83: 976–89. 14577825

[12] Pohl PS, McDowd JM, Filion DL, Richards LG, Stiers W (2001) Implicit learning of a perceptual-motor skill after stroke. Phys Ther 81: 1780–9. 11694171

[13] Meehan SK, Randhawa B, Wessel B, Boyd LA (2011) Implicit sequence-specific motor learning after subcortical stroke is associated with increased prefrontal brain activations: an fMRI study. Hum Brain Mapp 32: 290–303. 20725908 10.1002/hbm.21019 PMC3010500

[14] Yang Y, Hong-Yan B (2011) Unilateral implicit motor learning deficit in developmental dyslexia. Int J Psychol 46: 1–8. 22044127 10.1080/00207594.2010.509800

[15] van Tilborg IA, Kessels RP, Hulstijn W (2011) Learning by observation and guidance in patients with Alzheimer's dementia. NeuroRehabilitation 29: 295–304. 22142763 10.3233/NRE-2011-0705

[16] Gobel EW, Blomeke K, Zadikoff C, Simuni T, Weintraub S, et al (2013) Implicit perceptual-motor skill learning in mild cognitive impairment and Parkinson's disease. Neuropsychology 27: 314–21. 23688213 10.1037/a0032305 PMC4457378

[17] Halsband U, Lange RK (2006) Motor learning in man: a review of functional and clinical studies. J Physiol Paris 99: 414–24. 16730432 10.1016/j.jphysparis.2006.03.007

[18] Orrell AJ, Eves FF, Masters RSW (2006) Motor learning of a dynamic balancing task after stroke: implicit implications for stroke rehabilitation. Phys Ther 86: 369–80. 16506873

[19] Capio CM, Poolton JM, Sit CH, Eguia KF, Masters RSW (2013) Reduction of errors during practice facilitates fundamental movement skill learning in children with intellectual disabilities. J Intell Disabil Res 57: 295–305. 10.1111/j.1365-2788.2012.01535.x 22369034

[20] Lam WK, Maxwell JP, Masters RSW (2009) Analogy versus explicit learning of a modified basketball shooting task: performance and kinematic outcomes. J Sports Sci 27: 179–91. 19153868 10.1080/02640410802448764

[21] Maxwell JP, Masters RSW, Eves FF (2003) The role of working memory in motor learning and performance. Consc Cogn 12: 376–402. 10.1016/s1053-8100(03)00005-9 12941284

[22] Poolton JM, Masters RSW, Maxwell JP (2005) The relationship between initial errorless learning conditions and subsequent performance. Hum Mov Sci 24: 362–378. 16087262 10.1016/j.humov.2005.06.006

[23] Raab M, Masters RSW, Maxwell J, Arnold A, Schlapkohl N, et al (2009) Discovery learning in sports: Implicit or explicit processes? J Sports Sci 7: 413–30.

[24] Mount J, Pierce SR, Parker J, DiEgidio R, Woessner R, et al (2007) Trial and error versus errorless learning of functional skills in patients with acute stroke. NeuroRehabilitation 22: 123–32. 17656838

[25] Larin HM (2006) Motor learning: theories and strategies for the practitioner. In: Campbell SK, Vander Linden DW, Palisano RJ, editors. Physical Therapy for Children. 3rd ed. Philadelphia, PA: Saunders Elsevier.pp. 131–160.

[26] Linstone HA, Turoff M (1975) Introduction. In: Linstone HA, Turoff M, editors. The Delphi Method: Techniques and Applications. Reading, MA: Addison-Wesley Publishing Company. pp. 3–12.

[27] Kleynen M, Bleijlevens MH, Beurskens AJ, Rasquin SM, Halfens J, et al (2013) Terminology, taxonomy, and facilitation of motor learning in clinical practice: protocol of a delphi study. JMIR Res Protoc 2: e18. 23685621 10.2196/resprot.2604 PMC3668605

[28] Boulkedid R, Abdoul H, Loustau M, Sibony O, Alberti C (2011) Using and reporting the Delphi method for selecting healthcare quality indicators: a systematic review. PloS one 6: e20476. 21694759 10.1371/journal.pone.0020476 PMC3111406

[29] Powell C (2003) The Delphi technique: myths and realities. J Adv Nurs 41: 376–82. 12581103 10.1046/j.1365-2648.2003.02537.x

[30] Mokkink LB, Terwee CB, Knol DL, Stratford PW, Alonso J, et al (2006) Protocol of the COSMIN study: COnsensus-based Standards for the selection of health Measurement INstruments. BMC Med Res Methodol 6: 2. 16433905 10.1186/1471-2288-6-2 PMC1368990

[31] Steenbergen B, van der Kamp J, Verneau M, Jongbloed-Pereboom M, Masters RSW (2010) Implicit and explicit learning: applications from basic research to sports for individuals with impaired movement dynamics. Disabil Rehabil 32: 1509–16. 20575752 10.3109/09638288.2010.497035

[32] Schneider SA, Wilkinson L, Bhatia KP, Henley SM, Rothwell JC, et al (2010) Abnormal explicit but normal implicit sequence learning in premanifest and early Huntington's disease. Mov Disord 25: 1343–9. 20544716 10.1002/mds.22692 PMC2997693

[33] Lam WK, Maxwell JP, Masters RSW (2009) Analogy versus explicit learning of a modified basketball shooting task: Performance and kinematic outcomes. J Sports Sci 27: 179–91. 19153868 10.1080/02640410802448764

[34] Masters RSW, Lo CY, Maxwell JP, Patil NG (2008) Implicit motor learning in surgery: implications for multi-tasking. Surgery 143: 140–5. 18154942 10.1016/j.surg.2007.06.018

[35] Buchner A, Wippich W (1998) Differences and commonalities between implicit learning and implicit memory. In: Stadler MA, Frensch PA, editors. Handbook of Implicit Learning. Sage Publications, Thousand Oaks CA.

[36] Zafar SY, Currow DC, Cherny N, Strasser F, Fowler R, et al (2012) Consensus-based standards for best supportive care in clinical trials in advanced cancer. Lancet Oncol 13: e77–82. 22300862 10.1016/S1470-2045(11)70215-7

[37] Hasson F, Keeney S, McKenna H (2000) Research guidelines for the Delphi survey technique. J Adv Nurs 32(4): 1008–15. 11095242

[38] McCombe Waller S, Prettyman MG (2012) Arm training in standing also improves postural control in participants with chronic stroke. Gait Post 36: 419–24. 10.1016/j.gaitpost.2012.03.025 PMC3580767 22522046

